# Effect of Traditional Household Processes on Iron, Zinc and Copper Bioaccessibility in Black Bean (*Phaseolus vulgaris* L.)

**DOI:** 10.3390/foods7080123

**Published:** 2018-07-31

**Authors:** Sabrina Feitosa, Ralf Greiner, Ann-Katrin Meinhardt, Alexandra Müller, Deusdélia T. Almeida, Clemens Posten

**Affiliations:** 1 Department of Food Technology and Bioprocess Engineering, Max Rubner-Institut, Federal Research Institute of Nutrition and Food, Haid-und-Neu-Str. 9, D-76131 Karlsruhe, Germany; ralf.greiner@mri.bund.de (R.G.); ann-katrin.meinhardt@mri.bund.de (A.-K.M.); alexandra.mueller@mri.bund.de (A.M.); 2 School of Nutrition, Federal University of Bahia, Av. Araújo Pinho 32, Salvador 40110-150, Brazil; deliata@uol.com.br; 3 Institute of Life Science Engineering, Bioprocess Engineering, University of Karlsruhe, Fritz-Haber-Weg 2, 76131 Karlsruhe, Germany; clemens.posten@kit.edu

**Keywords:** beans, iron, zinc and copper bioaccessibility, *myo*-inositol phosphates, anti-nutrients, polyphenols, household processing

## Abstract

Micronutrient deficiencies are a major public health problem. Beans are an important plant-based source of iron, zinc and copper, but their absorption is reduced in the presence of anti-nutrients such as phytates, polyphenols and tannins. Soaking and discarding the soaking water before cooking is unanimously recommended, but this can result in mineral loss. Data on the consequences for mineral bioaccessibility is still limited. This study aimed to evaluate iron, zinc and copper bioaccessibility in black beans cooked (regular pan, pressure cooker) with and without the soaking water. For that, three batches of black beans were investigated in triplicate, each split in nine parts (raw grains and four different household processes in duplicate) and analyzed by applying the quarter technique, resulting in a grand total of 164 samples. Minerals were quantified by ICP-MS (inductively coupled plasma mass spectrometry), *myo*-inositol phosphates (InsP_5_, InsP_6_) by HPLC (high-performance liquid chromatography) ion-pair chromatography, total polyphenols using Folin-Denis reagent and condensed tannins using Vanillin assay. Mineral bioaccessibility was determined by in vitro digestion and dialysis. All treatments resulted in a statistically significant reduction of total polyphenols (30%) and condensed tannins (20%). Only when discarding the soaking water a loss of iron (6%) and copper (30%) was observed, and InsP_6_ was slightly decreased (7%) in one treatment. The bioaccessibility of iron and zinc were low (about 0.2% iron and 35% zinc), but copper presented high bioaccessibility (about 70%). Cooking beans under pressure without discarding the soaking water resulted in the highest bioaccessibility levels among all household procedures. Discarding the soaking water before cooking did not improve the nutritional quality of the beans.

## 1. Introduction

Deficiencies of micronutrients are a major public health problem, in which iron and zinc malnutrition affects more than half of the population worldwide [1,2]. Iron-deficiency anemia reaches more than 30% of the world’s population, approximately 20% in European Union and up to 40% in developing countries [1,3]. It contributes to 20% of maternal deaths besides being related to low adult productivity at work [3,4]. Outcomes of zinc deficiency are depressed growth, immune dysfunction, lower respiratory tract infections, diarrhea, altered cognition and other clinical conditions [4,5]. Copper deficiency may also lead to anemia, but features of human copper deficiency mechanisms are still unknown [6], while most copper research is focused on soil, fruits and nuts e.g., [7,8].

The major reason for iron deficiency is a poor availability of iron from the diet. Mineral deficiencies are not only caused by low dietary intake. Many other factors affect the absorption such as the total content of the minerals and anti-nutrients, the processing applied and mineral interactions [9,10]. The interactions concerning iron, zinc and copper appear to be especially important, and the bioaccessibility is influenced differently depending on the mineral [11,12]. Dietary and human factors, such as inflammation and disease, have been found to be the major factors influencing the bioavailability of micronutrients. Dietary factors are related to food matrix structure and composition, being mostly influenced by the interaction with other dietary compounds, such as fibers, lipids, proteins and anti-nutrients during digestion and absorption. It is also important to consider not only the total content of iron, zinc and copper in crops, but also the tissue localization (cotyledon and endosperm) and specification (chelates and protein particles) [11,13]. Iron exists in two different forms in food: hemic iron in animal products and non-hemic iron in plant foods which is generally poorly absorbed. Iron is stored in plants and animals as the protein ferritin, and about 80% of the iron in beans is present in the form of non-ferritin-bound iron which is possibly bound to *myo*-inositol phosphates [11,14]. Condensed tannins are able to form tannin-protein complexes that can chelate iron and calcium. Animal studies have demonstrated that in the presence of phytate, calcium can impair zinc absorption, probably by co-precipitation with phytate and zinc. Furthermore, digestibility and hence absorption of micronutrients such as iron and zinc can be improved upon heat processing, which results in softening of the food matrix, with release of protein-bound iron and zinc, thus facilitating its absorption. Studies in human subjects have shown that zinc may stimulate iron absorption, and calcium can inhibit iron absorption by inhibiting iron transport. Copper is essential for iron transport between tissues in which iron and copper homeostases are linked by the inability to export iron to the systemic circulation in the absence of copper. On the mechanistic level, neither zinc nor calcium seem to be as crucial for iron absorption as copper, but there are only few studies about copper deficiency and sufficient copper levels in the diet [6,11,12].

Beans are highly nutritious and the most consumed leguminous grain worldwide, which are an important plant-based source of iron, zinc and copper [15,16,17]. They are part of many traditional diets, playing a major role in vegetarian diets in all countries, besides being consumed in different dishes together with other food products [9,15,17,18]. Therefore, mineral bioavailability may also be influenced by interference with other food constituents [9,10]. Common beans are a staple food in Latin America and Eastern Africa [19,20] and Brazil is the most important consumer of beans in the world, with up to 19 kg/year per capita consumption, 80% of which is common bean and black bean is the second most consumed [17,21]. Approximately, a portion per meal of cooked beans (100 g) [15,17] contains 6.52–10.00 mg iron, 0.93–1.21 mg copper and 3.18–3.60 mg zinc, which equals the daily requirements for healthy adults for iron and copper and half of that of zinc (8 mg/day, 0.9 mg/day and 8–11 mg/day, respectively) [22]. Therefore, a regular intake of beans could contribute to minimize deficiencies of micronutrients [15,17]. The nutritional quality of beans, however, is usually reduced by the presence of anti-nutrients, such as phytates, polyphenols and tannins [9,20]. Those compounds bind to minerals such as iron, zinc, copper, calcium and magnesium, thus reducing bioavailability due to the formation of extremely insoluble salts or very poorly dissociated chelates.

Phytates (InsP_6_), have especially been reported to affect iron and zinc absorption negatively even at low concentrations [9,23,24,25]. Condensed tannins are able to form tannin-protein complexes, which can chelate iron and calcium [9,26,27]. A reduction of mineral bioavailability was observed when condensed tannins concentration was higher than 10% of the total dry weight of the samples or ranging from 2.5 to 4.7 mg eq. CE g^−1^ [27,28]. With regard to polyphenolic compounds, it has been reported that they reduce bioavailability of some minerals. Although there is no consensus on the quantity needed to decrease iron absorption in beans, a reduction in iron bioavailability was observed above 50 mg of polyphenols [27,29]. Furthermore, the polyphenols in legumes have been extensively correlated with health benefits in humans due to their potent anti-oxidant activities [30,31]. In common beans, those bioactive compounds mostly comprise phenolic acids and condensed tannins which are found in the cotyledons, and exhibit anti-diabetic, anti-obesity, anti-inflammatory, anti-mutagenic and anti-carcinogenic effects [30,31,32].

In a recent study [33], polyphenols of black beans were individually examined for their effect on iron uptake by Caco-2 cells. Half of the polyphenols studied were shown to inhibit iron absorption, but the other half were found to clearly promote iron absorption. So far, many studies [23,24,27,34] reported the link between a reduction of the total content of anti-nutrients in food grains with a higher availability of iron and zinc. Food processing and food preparation techniques like soaking, germination, hydrothermal treatment and fermentation can reduce the content of anti-nutrients [9,25,34]. Soaking and discarding the soaking water before cooking beans has been unanimously recommended due to a higher reduction of the anti-nutrients. An average reduction of 20% to 30% of condensed tannins and total polyphenols can be obtained in legumes by applying household processes [27,34,35]. The effect on mineral bioavailability was assessed in those studies mainly by molar ratios and statistical correlations between the content of anti-nutrients and the mineral content [27,34,35]. In general, digestibility and not bioavailability assays were applied in those studies. Bioavailability and bioaccessibility are often used indistinctly [36].

Only direct feeding trials can fully determine biological efficacy and mineral interactions, but they are long-lasting, cost intensive, and nonetheless the results need to be extrapolated to the human organism. A simple method to estimate the effect of for example food processing on mineral bioavailability is the use of bioaccessibility assays [19,24]. Although there is a substantial amount of information about binding of iron and zinc, and anti-nutrients reduction by food processing, data on the consequences for mineral absorption are still limited. Discarding the soaking water before cooking beans can result in loss of minerals and anti-oxidants and thus the nutritional quality of cooked beans is not necessarily improved. Thus, this study aimed to evaluate iron, zinc and copper bioaccessibility in black beans cooked with and without the soaking water using traditional household processes in order to expand knowledge about the nutritional value of this basic and accessible food and the options to use beans in combating micronutrients deficiencies.

## 2. Materials and Methods

All glassware used in sample preparation and analyses was washed in distilled water and for mineral analysis also immersed in a 5% nitric acid solution for more than 1 h and rinsed with ultrapure water (Milli-Q, Millipore, Merck KGaA, Darmstadt, Germany). The following describes in details the methods for analyzing three batches of black beans in triplicate, each split in nine parts (raw grains and four different household processes in duplicate), which were studied by applying the quarter technique, resulting in a total of 164 samples.

### 2.1. Samples

Three different batches of common beans (*Phaseolus vulgaris* L., black bean variety) from three randomly selected markets in Rio de Janeiro, Brazil, were used in this study. All batches were from commercial cultivation, geographic origin in the region of São Paulo, −23°10′45″ S, 45°53′12″ W, and harvested in June–July of 2015. The procedures applied during growth of the crop were not available. Moreover, the influence of the crop season on the black beans of this study is negligible [37]. The black bean samples were sent to Germany (Max Rubner-Institut, Karlsruhe, Germany), where the study (including the household processing) was performed in a period of one year, in a controlled environment to mitigate the influence of seasons to the experiment. The samples were stored at 4 °C with an extra vacuum-packaging. The raw grains were cleaned before use. All dirt was removed manually and then the beans were washed with deionized water. After that, the beans were cooked. For analyses the samples were freeze-dried (developed at the Max Rubner-Institut, Karlsruhe, Germany, operating with an air temperature of −30 °C and air velocity of 6 ms^−1^) and finely ground in a stainless steel analytical grinder (A10 Yellow Line, IKA-Werke GmbH & Co. KG, Staufen, Germany). Thereafter, a quarter technique was applied to the raw grains and the cooked beans together with the broth in order to obtain two final fractions properly homogenized. Each analytical determination was carried out in triplicate for each fraction of cooked and raw samples.

### 2.2. Household Treatments

In order to simulate traditional household processes for cooking beans, an overnight soaking (12 h) at room temperature was performed, followed by two cooking methods (boiling and pressure cooking) in tap water. Three different batches of black beans were used. A proportion of 100 g of the black beans and 400 mL of water were used for soaking. The following cooking strategies were performed: (1) with the soaking water in a pressure cooker; (2) without the soaking water in a pressure cooker; (3) with the soaking water in a regular pan; and (4) without the soaking water in a regular pan. The regular pan had a capacity of 3 L and the beans were cooked for 35 min. 200 mL of tap water were added during cooking to replenish the loss of evaporated water. The pressure cooker had a capacity of 3 L and the beans were cooked for 5 min. No water was added during the cooking process. The cooking times were chosen according to the results of a test cooking simulation. Before cooking the black beans, either tap water was added to the soaking water to give a final volume of 600 mL or the soaking water was discarded and replaced by tap water to give a final volume of 600 mL. All treatments were performed in duplicate for each batch of black beans. The same cooking methods were also performed without bean samples to quantify the concentrations of the minerals in the water before and after the cooking process.

### 2.3. Myo-Inositol Phosphates

Quantification of *myo*-inositol phosphates was performed by extracting 1 g of a freeze-dried sample with 20 mL of 2.4% HCl for 3 h with constant shaking at room temperature. The resulting suspensions were centrifuged (30 min, 15,000 rpm). The supernatant was collected and used for *myo*-inositol phosphate quantification [38]; 2 mL of the supernatant were diluted with ultrapure water to give a final volume of 60 mL. The entire solution was applied to a column (0.7 × 15 cm) containing 0.5 g of AG 1–X4 100–200-mesh resin (Bio-Rad Laboratories GmbH, München, Germany). The column was washed with 25 mL of ultrapure water and 25 mL of 25 mM HCl. Then *myo*-inositol phosphates were eluted with 25 mL of 2 M HCl. The eluates obtained were concentrated in a vacuum evaporator (Rotavapor RE-120, BÜCHI Labortechnik AG, Flawil, Switzerland) (at 40 °C) and dissolved in 1 mL of ultrapure water. Then 20 μL of the samples were chromatographed on Ultrasep ES 100 RP18 (2 × 250 mm). The column was run at 40 °C and 0.2 mL min^−1^ of an eluent consisting of formic acid/methanol/water/tetrabutylammonium hydroxide (44:56:5:1.5 *v*/*v*), pH 4.25. A mixture of the individual *myo*-inositol phosphate esters (InsP_3_–InsP_6_) was used as a standard [39]. The retention times of InsP_5_ and InsP_6_ were 15 min and 23 min, respectively.

### 2.4. Total Polyphenols

Total phenols were extracted with water. An internal standard curve was prepared by adding 10 mL of 0–0.01% tannic acid to the flasks. The flasks were heated for 30 min at 70 °C with constant shaking. Clear supernatants were collected after centrifugation at 2500 *g* for 15 min followed by filtration. Polyphenols were determined using the Folin–Denis reagent [40].

### 2.5. Condensed Tannins

Condensed tannins were extracted with HCl:methanol (1:100 *v*/*v*) for 2 h with mechanical shaking (Universal shaker SM, Carl Roth GmbH + Co. KG, Karlsruhe, Germany) at 25 °C and centrifuged (Sorvall LYNX 6000, Thermo Scientific, Langenselbold, Germany) at 5000 *g* at 15 °C for 15 min. Aliquots were immediately analyzed for tannins using the 0.5% vanillin assay [41].

### 2.6. Minerals

Iron (Fe), zinc (Zn), copper (Cu) and calcium (Ca) concentrations were measured. Therefore, 150 mg of each ground sample was microwave-digested in a MWS–1 (Berghof Products + Instruments GmbH, Eningen, Germany) with 3 mL of concentrated HNO_3_ (65% *v*/*v*) and 0.75 mL of H_2_O_2_ (30% *v*/*v*). Heating was performed in four successive steps: linear temperature increased up to 150 °C in 5 min (80 W); 5 min at 150 °C (70 W); linear temperature increased up to 180 °C in 40 min (80 W); 10 min at 180 °C (80 W). All samples were analyzed in triplicate and a set of digestion blanks were prepared with each sample batch. The data was expressed as mean ± standard deviation on dry matter (DM) basis.

Element analysis was performed by inductively coupled plasma mass spectrometry (ICP-MS), iCAP Q (Thermo Scientific, Waltham, MA, USA). The ICP-MS operating conditions and measurement parameters are given in Table 1. Standard addition was used for calibration. The limit of quantification (LOQ) was calculated based on the measured values of the blanks (*n* = 152), where LOQ = mean + 10× standard deviation. The extreme studentized deviate test was used to remove outliers from the data set. Fresh kidney beans NCS ZC73019 (GSB–12) was used as reference material (*n* = 84) to determine precision and accuracy of the method (Table 2). The relative standard deviations were less than 3% for all investigated elements, and at a 95% confidence level showed that there was no significant difference between the means of the certified and determined values for the analytes under investigation.

### 2.7. Iron, Zinc and Copper Bioaccessibility

In order to be able to quantify bioaccessibility in cooked black bean samples, a simplified *in vitro* gastrointestinal digestion assay was carried out. Iron, zinc and copper bioaccessibilities were determined based on *in vitro* digestion and dialysis method described by [42] with modifications. For gastric digestion, 10 g of ground sample were suspended in 60 mL of 20 mM glycine-HCl buffer, pH 2.0. After, adjusting pH to 2.0 by with 2 M HCl, 1.3 mL of pepsin (porcine, Fluka Analytical, Sigma-Aldrich Chemie GmbH, Steinheim, Germany) solution (1.6 g pepsin in 10 mL 20 mM glycine-HCl buffer, pH 2.0) were added. The suspension was incubated at 37 °C for 2 h under agitation. To simulate intestinal digestion, the pH of the gastric digestion was adjusted to 7.2 with 1 M NaHCO_3_. 13 mL of a pancreatin (porcine, P1750, Sigma-Aldrich Chemie GmbH, Steinheim, Germany) solution (0.4 g pancreatin in 100 mL of ultrapure water) were added and a dialysis bag (cut of 10,000 Da; Carl Roth GmbH + Co. KG, Karlsruhe, Germany, containing 2 mL of ultrapure water) was placed in the digestion system. The system was incubated at 37 °C for 2 h, under agitation. Thereafter, the dialysis bag was removed and iron, zinc and copper in the dialysate were analyzed by ICP-MS. Bioaccessibility (%) was calculated as 100 × Y/Z whereby Y represents the dialyzable amount of the mineral per 100 g DM of cooked beans and Z the total of the same mineral per 100 g DM of the cooked beans.

### 2.8. Statistical Analysis

All the analyses were conducted in triplicate and expressed as mean ± standard deviation of three separate determinations. The results were evaluated for normality by the Shapiro–Wilk test. The data generated was subjected to one-way analysis of variance (ANOVA) using the software Sigma Plot version 13.0. A Tukey’s paired comparison test was used to determine statistically significant differences (*p* < 0.05) among the batches and in between raw and treated samples mean values, at a 95% confidence level.

## 3. Results and Discussion

### 3.1. Mineral Contents of Raw and Cooked Beans

**Figure 1:**
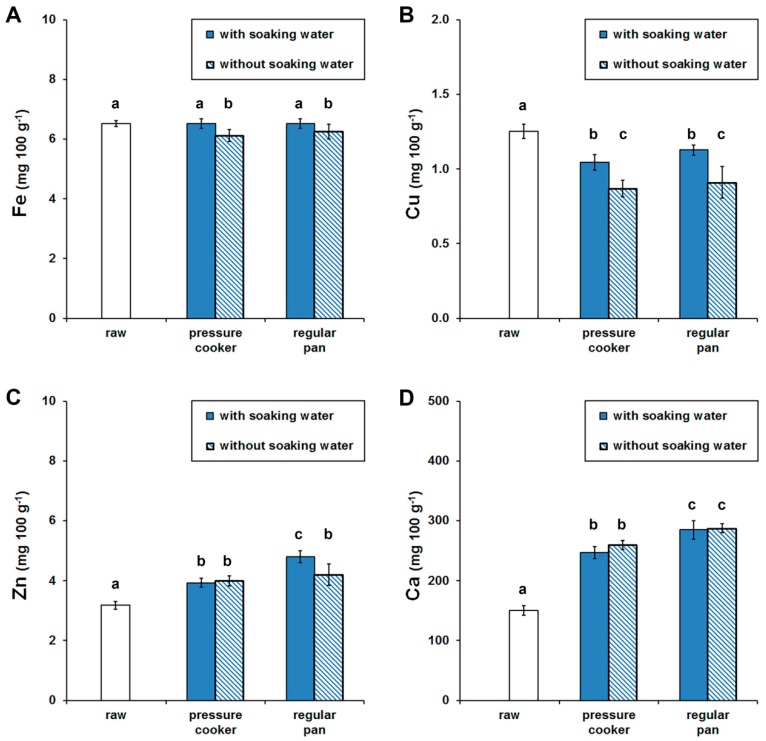
Black bean contents of iron (**A**), copper (**B**), zinc (**C**) and calcium (**D**). Data expressed as mean ± standard deviation (dry matter). Values marked by different letters are significantly different (*p* < 0.05).

The mean iron, zinc, copper and calcium contents of the three black bean batches are presented in Figure 1. All batches were not significantly different (*p* > 0.05) among each other.

Black beans were confirmed to be a good source of iron, zinc and copper. Approximately a portion per meal of cooked black beans (100 g) contains an average of 6.5 mg iron, 4 mg zinc and 1 mg copper. Those contents are in good agreement with data published by the Food and Agriculture Organization of the United Nations database [15] for common beans of the same origin. Thus, 100 g of black bean meets the daily requirement for copper (0.9 mg/day), and partially that for iron (8 mg/day) and zinc (8–11 mg/day) [22].

Discarding the soaking water before cooking the beans resulted in a lower content (*p* < 0.001) of iron (6%) and copper (30%) compared to the raw beans (Figure 1A,B). According to Raes et al. [13], the differences in leaching of micronutrients can be attributed to the fact that these minerals are bound by different food constituents with different binding strength. Furthermore, their location within the food matrix might be different. Zinc and calcium contents was found to be increased irrespective of the household procedure applied (*p* < 0.001). The highest contents (Zn: 132–150%, Ca: 191%) were found in black beans cooked in a regular pan (Figure 1C,D).

The concentrations of the minerals in the water before and after the cooking process were measured and hence either present in the tap water or released from the pressure cooker or pan. Iron and copper concentrations of the tap water before and after cooking were below the LOQ (mg 100 g^−1^): Fe (0.14), Cu (0.56). Zinc mean concentrations were determined to be below the LOQ (0.51 mg 100 mL^−1^) in the tap water. In the boiled water samples, it ranged from 0.75 ± 0.06 mg (pressure cooker) to 1.92 ± 0.32 mg (regular pan). Therefore, the increase in the Zn contents was found to be due to a leaching of zinc ions from the pan surface. A smaller increase (*p* < 0.05) in Zn content (123–125%) was also observed during pressure cooking (Figure 1C). Katzenberg et al. [43], also reported higher zinc concentrations in beef as an effect of the cooking method and Quintaes et al. [44], have shown the migration of metal ions from cookware into foods.

The mean Ca concentration in the tap water was determined to be 16.83 ± 1.38 mg 100 mL^−1^. Thus, the theoretical amount of calcium added was 100.98 mg 100 g^−1^ for pressure cooking and 134.64 mg 100 g^−1^ in the regular pan. The observed increases in calcium were found to be 97.00 ± 1.57 mg 100 g^−1^ (with soaking water) and 109.27 ± 0.61 mg 100 g^−1^ (without soaking water) for pressure cooking, and 134.66 ± 7.23 mg 100 g^−1^ (with soaking water) and 136.95 ± 0.80 mg 100 g^−1^ (without soaking water) using the regular pan (Figure 1D). Therefore, the increase in Ca contents was due to the addition of tap water during cooking.

### 3.2. Anti-Nutrients

**Figure 2:**
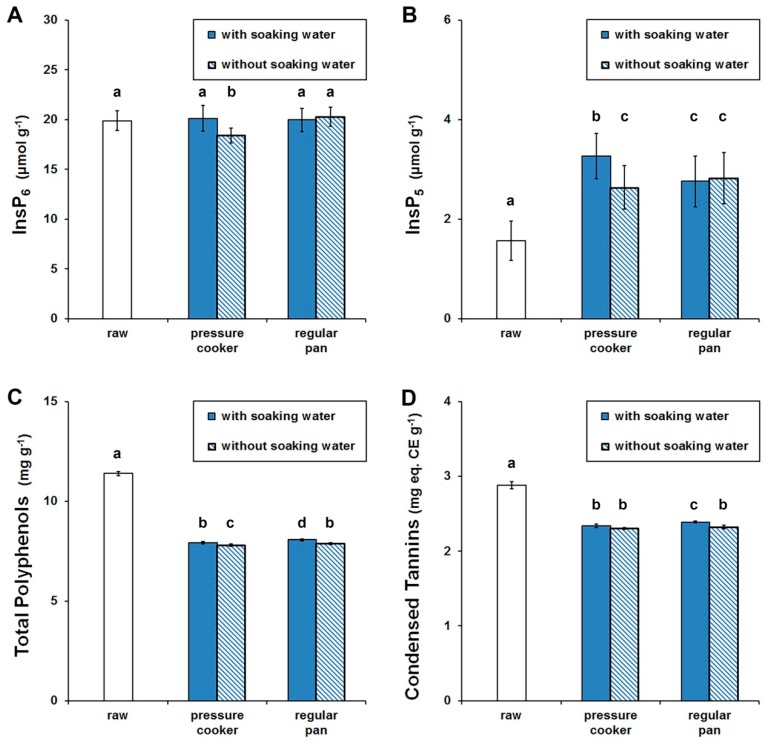
Black bean anti-nutrients content of InsP_6_ (**A**), InsP_5_ (**B**), total polyphenols (**C**) and condensed tannins (**D**). Data expressed as mean ± standard deviation (dry matter). Values marked by different letters are significantly different (*p* < 0.05).

The mean contents of InsP_6_, InsP_5_, total polyphenols and condensed tannins are presented in Figure 2. All traditional household processes applied resulted in a statistically significant reduction in total polyphenols (about 30%) and condensed tannins (about 20%) compared to raw black bean (Figure 2C,D). Discarding the soaking water before cooking the beans resulted in a greater reduction of polyphenols and tannins, which is in good accordance with the majority of studies [27,34]. Since polyphenols of legumes have been extensively correlated with health benefits in humans due to their potent anti-oxidant activities [30,31,32], their reduction during processing does not necessarily improve the nutritional quality of beans. With regard to *myo*-inositol phosphates, only with beans cooked without the soaking water in a pressure cooker a slightly decrease (7%) in InsP_6_ content was observed. InsP_5_ contents, however, increased with all cooking procedures applied. Furthermore, no statistical difference was observed among the three batches of the black bean samples regarding the contents of anti-nutrients.

### 3.3. Bioaccessibility of Iron, Zinc and Copper

The mean levels (%) of iron, zinc and copper bioaccessibility in cooked black beans are shown in Table 3. The determination of the micronutrient bioaccessibility makes it possible to estimate the percentage of absorption of those minerals with a simple and affordable assay compared to bioavailability assessment. Beans are highly nutritious legumes that have been reported as one of the best plant-based sources of bioaccessible iron and zinc [18,19,20]. In this study however, iron bioaccessibility levels were found to be low with all household processes (Table 3). On the other hand, copper showed high bioaccessibility, followed by zinc (Table 3).

Recent reviews [27,34] reported a link between iron and zinc availability from common beans and cooking soaked beans without the soaking water. This was reported to be due to the reduction of the content in anti-nutrients during food processing [9]. In this present study, however, black bean cooked with the soaking water in a pressure cooker resulted in the highest bioaccessibility for all three minerals in spite of higher total anti-nutrients reduction in beans cooked without the soaking water. According to Hoppler et al. [14], 70–85% of the iron in beans is present in the form of non-ferritin-bound iron and it is possibly bound to *myo*-inositol phosphates. Phytate is abundant in legumes, cereals and nuts, being considered to be the most powerful anti-nutrient due to their high binding capacity for metals and also their ability to form large insoluble aggregates [23,25].

Discarding the soaking water was shown to have a negative effect on the bioaccessibility of all three minerals in regular pan. Assessing mineral bioavailability in those studies mainly by molar ratios and statistical correlations between the content of anti-nutrients and the mineral content might be responsible for the observed differences in the obtained results [27,34,35]. In addition, details on the cooking methods applied were not reported. Pereira et al. [19], studied the effect of household cooking methods on the bioaccessibility of iron and zinc in different beans cultivars. Iron bioaccessibility of beans cooked with the soaking water in a pressure cooker were higher (6.46–40.68%) compared to beans cooked in a regular pan (2.42–8.92%). In the present study, the same tendency was observed. Even if lower Fe bioaccessibilities were found in this study, Zn bioaccessibilities were observed to be always higher than Fe bioaccessibilities. Bioaccessibility studies are a useful method to estimate the general trend of a household procedure on mineral bioavailability, but the absolute data obtained in those studies do not represent the situation in a human digestive tract. Neither active mineral uptake nor the interaction of minerals with respect to binding to food constituents or interaction with mineral transporters in the small intestine can be considered through bioaccessibility studies. The interactions concerning iron, zinc and copper appear to be of utmost importance in respect to their bioavailability [11,12]. Since micronutrient uptake has been successfully studied by Caco-2 cells models due to their exclusive ability to model human absorption characteristics [45,46,47], the data obtained in this study should be confirmed using a Caco-2 cell model.

## 4. Conclusions

Black beans were confirmed to be a good source of iron, zinc and copper with a high bioaccessibility of copper (about 70%) from cooked beans. The bioaccessibility of iron and zinc, however, were found to be low (about 0.2% for iron and 35% for zinc). Cooking beans under pressure without discarding the soaking water resulted in the highest bioaccessibility levels among all household procedures applied. Although a reduction in anti-nutrients’ content was observed, the *myo*-inositol phosphate content did not change significantly. In addition, discarding the soaking water before cooking the beans did not improve their nutritional quality. This procedure resulted in a loss of iron, copper and bioactive compounds.

Data on the consequences for iron, zinc and copper absorption are still limited. Thus, improving knowledge about the influence of traditional household processes on the nutritional value of this basic and accessible food is important. Further work is necessary to increase especially iron availability in home-cooked beans. Since phytate is the constituent with the highest impact on mineral bioavailability in common beans, applying the most efficient household procedures combined with phytase application might be a promising approach.

## Figures and Tables

**Table 1:** ICP-MS operating conditions and measurement parameters.

Parameter	Value
Radiofrequency power	1550 W
Argon flow rates	
Cooling	13.8 L min^−1^
Auxiliary	0.65 L min^−1^
Nebulizer	1.05 L min^−1^
Sample cone	Ni
Skimmer cone	Ni
Analyte	43Ca, 56Fe, 65Cu, 66Zn
Internal standard	103Rh (Fe, Cu, Zn), 45Sc (Ca, Fe), 89Y (Fe, Cu), 72Ge (Ca, Zn), 115In (Cu, Zn)
Aquisition/scanning mode	STD (Ca), KED (Fe, Cu, Zn)
Sweeps per reading	100
Dwell time	10 ms (Ca, Cu, Zn); 40 ms (Fe)
No. of runs	5
Replicate time	21 s
Sample uptake rate	0.2 mL min^−1^
Wash time between samples (2% HNO_3_)	30 s
Sample delay	50 s
Stabilization time	5 s

ICP-MS: Inductively Coupled Plasma Mass Spectrometry.

**Table 2:** ICP-MS precision and accuracy of the method.

Element	LOQ (µg kg^−1^)	Reference Material Measured Value (mg kg^−1^)	Reference Material Certificate Value (mg kg^−1^)
Ca	29.8	0.66 ± 0.06	0.67 ± 0.04
Fe	1.8	306 ± 29	330 ± 20
Cu	7.2	8.4 ± 1.5	8.7 ± 0.5
Zn	6.6	34 ± 4	32 ± 2

LOQ: limit of quantification.

**Table 3:** Bioaccessibility levels (%) of iron, zinc and copper in black bean cooked with traditional household processes.

Household Processes	Iron (%)	Zinc (%)	Copper (%)
Regular pan with soaking water	0.18 ^a^	33.94 ^a^	71.53 ^a^
Pressure cooker with soaking water	0.33 ^b^	44.66 ^b^	73.35 ^a^
Regular pan without soaking water	0.17 ^a^	31.55 ^a^	66.42 ^b^
Pressure cooker without soaking water	0.22 ^a^	35.04 ^a^	68.16 ^b^

In each column, values marked by different letters are a significantly different (*p* < 0.05).

## Limitations

Although this study was carefully conducted and has reached its aim, there was an unavoidable limitation. The specified information regarding what procedures were applied during growth of the crop is not available for the commercial cultivation of black beans used in this research. In addition, to mitigate the influence of seasons when the study was conducted, the experiments were performed entirely in a controlled environment.
